# Combined impact of lifestyle-related factors on total mortality among the elder Chinese: a prospective cohort study

**DOI:** 10.1186/s12877-022-02982-z

**Published:** 2022-04-14

**Authors:** Zhiwei Lian, Chunsu Zhu, Haowen Yuan, Ying Chen

**Affiliations:** 1 Fujian Medical University Cancer Hospital, Fujian Cancer Hospital, No. 420, Fuma Road, Jinan District, Fuzhou, 350014 China; 2 School of Public Health, Peking University, 100191 Beijing, China

**Keywords:** Healthy lifestyle, Mortality, Seniors, Cohort study

## Abstract

**Background:**

The combined impact of healthy lifestyle factors on total mortality among elder Chinese is unclear. This study aimed to investigate the overall impact of lifestyle factors on total mortality in a senior Chinese population, and determine whether these associations were consistent in the presence of different characteristics, including physical comorbidities.

**Methods:**

The Chinese Longitudinal Healthy Longevity Survey (CLHLS) is a large population-based prospective cohort study in 22 of 31 provinces from mainland China. We included 15,163 adults aged ≥65 years recruited from 1998- to 2002 and followed-up until 2014. A healthy lifestyle score was calculated considering five lifestyle factors (exercise, smoking, dietary diversity, body mass index and drinking). The scores ranged from zero to five points and were classified into the following three categories: unhealthy (0-1 point), intermediate (2-3 points) and healthy (4-5 points). Cox proportional hazards regression analyses were used to assess the associations between the combined healthy lifestyle score and total mortality, adjusting for demographic characteristics and physical comorbidities, as appropriate. Stratification analyses and interaction analyses were further performed.

**Results:**

Among the 15,163 participants, the mean age (SD) was 86.2 (11.6) years. During an average follow-up period of 12.5 (SD = 3.9) years, 9655 deaths occurred. The adjusted hazard ratios (HRs) of total mortality decreased as the number of healthy lifestyle factors increased. Compared to the unhealthy lifestyle group, the healthy lifestyle group had a HR and 95% CI of 0.78 and 0.72-0.83. The population attributable risk of total death among those without a healthy lifestyle was 25.2%. A healthier lifestyle pattern was associated with a lower total mortality risk among individuals with different severities of physical comorbidities, although the associations were stronger among those with fatal physical comorbidities (*p*_*-interaction*_ < .001).

**Conclusions:**

In this large-scale study, a healthier lifestyle measured by regular exercise participation, never smoking, never drinking, good dietary diversity and normal weight, was inversely associated with total mortality, regardless of physical comorbidity status. These findings support the necessity of multiple lifestyle modifications to prevent premature death in both general elderly populations and those with physical comorbidities.

**Supplementary Information:**

The online version contains supplementary material available at 10.1186/s12877-022-02982-z.

## Introduction

Population aging is a current global phenomenon in both developed and developing countries [1, 2]. In China, a large explosion in the elderly population is expected in 2050, with an estimate of 26.1% of Chinese citizens aged older than 65 years [3–5]. Given the large size of the elderly population, increasing emphasis should be placed on the aging process. Previous studies have suggested that lifestyle factors, including smoking, drinking, exercise, diet and body mass index (BMI), are closely associated with the occurrence of various non-communicable diseases (NCDs) and preventable deaths [6, 7]. For instance, it was estimated that unhealthy lifestyle caused more than 23 million deaths and 36.5% of disability-adjusted life years worldwide in 2017 [8]. Fortunately, these lifestyle factors are modifiable, and promoting healthy lifestyle-oriented strategies might be the most cost-effective method in preventing NCDs and deaths, especially in low- and middle-income countries, where resources are limited [9]. Moreover, since these lifestyle factors often have synergistic and complementary effects on the development of NCDs and deaths [10], it is necessary to assess the contribution of multiple lifestyle factors jointly.

Recently, the combined impact of lifestyle-related factors on total deaths has attracted much attention, and understanding the relationships between combined lifestyle factors and mortality is of vital importance for medical resource allocation and health policy development. Previous studies on lifestyle factors and all-cause mortality have been conducted primarily in Western populations [11, 12]; few studies were conducted in other populations, including Chinese populations, with limitations [13–16]. The findings from Western populations may not apply to the Chinese population because of different lifestyle customs and diverse disease spectra. In China, many women do not actively smoke or consume alcohol regularly [17], physical activity is mainly derived from occupational work and housework [18], and stroke is the leading cause of death [19]. In addition, evidence in the elderly population has been limited, as previous studies often had small sample sizes and short follow-up periods and did not comprehensively evaluate whether these associations were consistent among individuals with different characteristics (e.g., participants with potentially fatal diseases at baseline versus those without) [20, 21]. To the best of our knowledge, no nationally representative study investigating the relationships between combined lifestyle factors and total death among elderly people has been conducted in China to date. Although adopting a healthy lifestyle may decrease the incidence and mortality of NCDs, such as stroke, whether and the extent to which accumulated lifestyle factors impact all-cause mortality in senior individuals is unclear.

Therefore, in the present study, we aimed to 1) examine the individual and combined impacts of lifestyle factors on the risk of total mortality among the Chinese population aged ≥65 years and 2) clarify whether these associations are consistent in the presence of different characteristics, including physical comorbidities, at baseline.

## Methods

This study followed the STrengthening the Reporting of OBservational Studies in Epidemiology (STROBE) guidelines [22].

### Study population

The data used in this study were derived from the Chinese Longitudinal Healthy Longevity Survey (CLHLS), a nationwide, community-based, longitudinal cohort study. A detailed description of the CLHLS has been provided previously [23]. Briefly, the survey began in 1998, and subsequent follow-up surveys were carried out in 2000, 2002, 2005, 2008, 2011, and 2014, with an estimated response rate of 90% in each wave. To ensure the representativeness of CLHLS, the multi-stage cluster sampling approach was utilized in this project. In total, 22 of China’s 31 provinces were initially selected as primary survey units, and then, half of the cities or counties in these provinces were randomly selected. In total, 631 cities or counties in mainland China were selected, with a target sample covering 85% of China’s total population. Information concerning the sociodemographic status, lifestyle, and physical health was collected by well-trained interviewers using a structured questionnaire.

In the present study, data from 5 waves of the CLHLS from 2002 to 2014 were included and individuals aged over 65 years were excluded until 2002. In total, 16,064 individuals were interviewed in 2002, and we excluded participants for the following reasons: missing data related to lifestyle factors (*n* = 665, 4.1%), sociodemographic characteristics (*n* = 132, 0.8%) and NCDs (*n* = 35, 0.2%) and loss to follow-up shortly after the baseline recruitment (*n* = 69, 0.4%). Overall, few data for lifestyle factors were missing (< 5%), and less than 1% of the data for other variables were missing. Thus, we did not impute the missing data. The final sample of 15,163 participants aged ≥65 years was selected.

### Healthy lifestyles

We included five lifestyle-related factors (dietary diversity, smoking, exercise, drinking, and BMI). The food consumption data were collected at baseline based on a food frequency questionnaire including seven major food groups—fruits, vegetables, meat, fish, eggs, beans and tea. The responses for each food group were recorded as “often of almost every day” or “occasionally” or “rarely or never”. Individuals with responses of “often or almost every day” received a score of 1; the others received a score of 0. The total dietary diversity score was calculated by summing the scores across all items, for a maximum score of 7, with a higher score indicating better dietary diversity. As used in previous studies [24, 25], dietary diversity was defined as ‘good’ if the dietary diversity score was at or above the mean value. During the baseline interview, the participants were asked the question “Do you do exercise regularly at present, including jogging, playing ball, running or Qigong?” and the responses were recorded as yes or no. The smoking status (never smoker, former smoker, current-smoker) and alcohol consumption (never, former, and current) were assessed by self-report at baseline. The participants who never smoked, never drank and exercised regularly were classified as healthy. Height and weight were measured to calculated the BMI as bodyweight (kg) divided by the squared body height (m^2^). To minimize reverse causality, lifelong maximum BMI was used in this study [26, 27]. BMI was classified into four categories according to the guidelines for Chinese people as follows: underweight (< 18.5 kg/m^2^), normal weight (≥ 18.5 kg/m^2^ and < 24.0 kg/m^2^), overweight (≥ 24.0 kg/m^2^ and < 28.0 kg/m^2^) and obese (≥ 28.0 kg/m^2^). Individuals with normal weight were classified as healthy.

The healthy lifestyle score was calculated based on previous research [20, 21, 28]. For each lifestyle behavior, the participant received a score of 1 if the subjects fulfilled the criteria for health or 0 if they did not (Table 1). The total healthy lifestyle score was calculated by summing the points of each lifestyle factor, contributing to a final lifestyle score ranging from 0 to 5, with higher scores indicating a healthier lifestyle. The scores were classified into the following three lifestyle groups as the sample sizes of zero and five points were relatively small: unhealthy (0-1 factors), intermediate (2-3 factors) and healthy (4-5 factors) [21].

**Table 1:** Combined healthy lifestyle scores in the Chinese longitudinal healthy longevity survey

Lifestyle Factors	Classification	Scoring Classification
BMI (kg/m2)	Underweight, < 18.5	0
	Normal weight, 18.5 ≤ BMI < 24.0	1
	Overweight, 24.0 ≤ BMI < 28.0	0
	Obese, BMI ≥ 28.0	0
Smoking status	Never smoker	1
	Former smoker	0
	Current smoker	0
Alcohol consumption	Never	1
	Former	0
	Current	0
Dietary diversity	Below average score, < 2.5	0
	Average score and above, ≥2.5	1
Regular exercise participation	Yes	1
	No	0

### Assessment of mortality

In the follow-up surveys in 2005, 2008, 2011, and 2014, information concerning the survival status and date of death was collected through interviews with close family members or village doctors. A ‘loss to follow-up’ was defined as an individual who could not be reached or contacted after more than three reasonable efforts. The participants who survived in the 2014 wave survey or who were lost-to follow-up were defined as censored. Those who were lost-to follow-up in the follow-up surveys were included in the analysis. The person years were calculated from baseline until the date of death, loss to follow-up (3909, 25.8%), or the date of the last follow-up in 2014, whichever occurred first.

### Assessment of covariates

Covariates were obtained by a structured questionnaire at baseline. One section included demographic characteristics, containing details of age, sex, education level, place of residence and marital status. The other section dealt with self-reported NCDs diagnosed by a doctor, comprising hypertension, diabetes, stroke and other cerebrovascular diseases, heart disease and cancer. Education level was classified as illiteracy and literacy. Marital status was categorized as “married” if an individual was currently married, and “others” if an individual had never married or was widowed or divorced. Place of residence was classified as urban (those living in city or town) and rural (those living in the countryside).

### Statistical analysis

The distributions of the baseline characteristics were analyzed according to the lifestyle categories. The continuous variables were presented as means and standard deviation (SD), and the categorical variables were shown as absolute and relative frequencies. Chi-squared test was used to compare the categorical variables of different lifestyle groups.

Cox proportional hazard regression analyses were carried out to calculate the hazards ratio (HR) and 95% confidence interval (CI) of each lifestyle factor. All potential confounding factors including age (continuous variable), sex, education level, marital status, residence, physical comorbidities and other lifestyle factors were adjusted, as appropriate. Cox proportional hazard regression models were also used to assess the association between the combined healthy lifestyle and total mortality, using an unhealthy lifestyle (score 0-1) as the referent. We first controlled for age and sex (model 1), and then, the other demographic factors were added (model 2). In the fully adjusted model, we additionally adjusted for NCDs (model 3). The linear trends were evaluated using the Wald test, with the lifestyle score as a continuous variable, and adjustment for demographic factors and physical comorbidities was performed. We assessed the proportional hazards assumption graphically and found no evidence of obvious departure from this assumption.

To quantify the contribution of healthy lifestyle factors to the burden of total mortality, we calculated the total population attributable risk (PAR %), which is an estimation of the percentage of mortality in the population that theoretically would not have occurred if all individuals adopted a healthy lifestyle based on the assumption that the observed associations between lifestyle factors and mortality were causal [29, 30]. In these analyses, we introduced an approach applicable to multi-category exposures that had been proposed previously [13, 30]. We first estimated the PARs of the specific categories in relation to the highest score, i.e., two to three healthy lifestyle factors compared with four to five factors. Then, the total PAR was calculated by summing these specific PARs. To test the robustness of the findings, all analyses of the different lifestyle categories were repeated under the following scenarios: (1) exclusion of those aged over 85 years at baseline, (2) exclusion of those who died within the first year of follow-up, (3) exclusion of those with potentially fatal diseases, including heart disease, cancer or cerebrovascular disease, and (4) exclusion of those who were underweight (BMI <  18.5 kg/m^2^). Trichopoulou et al. previously described an approach to assess the relative importance of each of the five components of the lifestyle score on mortality [31]. One factor at a time from the original lifestyle score was alternately excluded, and the 5 hazards ratios (HRs) in association with a 1-unit increase in the score were analyzed.

Stratification analyses considering all the aforementioned covariates were carried out, and the chi-squared-based Q test was used to assess heterogeneity [32]. Interaction analyses were further conducted by entering multiplicative terms into the fully adjusted models. We also assessed these associations among the following three subgroups classified by the severity of physical comorbidities at baseline: (1) elder participants with potentially fatal chronic diseases, including heart disease, cancer and cerebrovascular disease (*n* = 3128); (2) participants with less serious chronic diseases, including hypertension and diabetes (*n* = 1680); (3) participants with no history of the aforementioned conditions (*n* = 10,265). We used the Statistical Package for the Social Sciences (SPSS) version 21.0 to perform the analyses, and R software 3.6.1 to draw the figures. A two-tailed *P* < .05 was regarded as statistically significant.

## Results

Of the 15,163 participants, the mean (SD) age at baseline was 86.2 (11.6) years, and 8684 (57.3%) were women (Table 2). Women were more likely than men to live a healthy lifestyle. As the number of lifestyle factors increased, the individuals tended to be younger, more educated, and less likely to suffer from hypertension, diabetes, heart disease, cerebrovascular disease and cancer (all *P* < .001) (Table 2).

**Table 2:** Baseline characteristics of 15,163 Chinese older participants by lifestyle category

Characteristics	Participants, No. (%)				***P*** value
	Total sample (***n*** = 15,163)	Unhealthy lifestyle (***n*** = 2056 [13.6%])	Intermediate lifestyle (***n*** = 9988[65.9%])	Healthy lifestyle (***n*** = 3119[20.5%])	
Age, y					<.001
65-79	4658(30.7)	557(27.1)	2915(29.2)	1186(38.0)	
80-94	6258(41.3)	881(42.9)	4092(41.0)	1285(41.2)	
> 95	4247(28.0)	618(30.0)	2981(29.8)	648(20.8)	
Sex					<.001
Men	6479(42.7)	1121(54.5)	3866(38.7)	1492(47.8)	
Women	8684(57.3)	935(45.5)	6122(61.3)	1627(52.2)	
Education					<.001
Illiteracy	9392(61.9)	1300(63.2)	6586(65.9)	1506(48.3)	
Literacy	5771(38.1)	756(36.8)	3402(34.1)	1613(51.7)	
Region					<.001
Urban	6940(45.8)	706(34.3)	4306(43.1)	1928(61.8)	
Rural	8223(54.2)	1350(65.7)	5682(56.9)	1191(38.2)	
Marital status					<.001
Married	4521(29.8)	573(27.9)	2757(27.6)	1191(38.2)	
Widowed and others	10,642(70.2)	1483(72.1)	7231(72.4)	1928(61.8)	
Smoking status					<.001
Never	9983(65.8)	447(21.7)	6735(67.4)	2801(89.8)	
Former	2709(17.9)	830(40.4)	1709(17.1)	170(5.5)	
Current	2471(16.3)	779(37.9)	1544(15.5)	148(4.7)	
Alcohol consumption					<.001
Never	10,111(66.7)	308(15.0)	6923(69.3)	2880(92.3)	
Former	2422(16.0)	854(41.5)	1460(14.6)	108(3.5)	
Current	2630(17.3)	894(43.5)	1605(16.1)	131(4.2)	
Dietary diversity					<.001
Poor	6851(45.2)	1731(84.2)	4869(48.7)	251(8.0)	
Good	8312(54.8)	325(15.8)	5119(51.3)	2868(92.0)	
BMI, kg/m^2^					<.001
< 18.5	6580(43.3)	538(26.2)	3791(38.0)	2251(72.2)	
18.5- 23.9	131(0.9)	38(1.8)	79(0.8)	14(0.4)	
24.0-27.9	6370(42.0)	1177(57.2)	4525(45.3)	668(21.4)	
≥ 28.0	2082(13.7)	303(14.7)	1593(15.9)	186(6.0)	
Regular exercise					<.001
No	10,318(68.0)	1944(94.6)	7566(75.8)	808(25.9)	
Yes	4845(32.0)	112(5.4)	2422(24.2)	2311(74.1)	
Hypertension					<.001
No	11,372(75.0)	1655(80.5)	7530(75.4)	2187(70.1)	
Yes	3791(25.0)	401(19.5)	2458(24.6)	932(29.9)	
Diabetes					<.001
No	13,231(87.3)	1865(90.7)	8747(87.6)	2619(84.0)	
Yes	1932(12.7)	191(9.3)	1241(12.4)	500(16.0)	
Heart disease					<.001
No	12,459(82.2)	1788(87.0)	8269(82.8)	2402(77.0)	
Yes	2704(17.8)	268(13.0)	1719(17.2)	717(23.0)	
Cerebrovascular disease					<.001
No	13,077(86.2)	1821(88.6)	8653(86.6)	2603(83.5)	
Yes	2086(13.8)	235(11.4)	1335(13.4)	516(16.5)	
Cancer					<.001
No	13,843(91.3)	1926(93.7)	9149(91.6)	2768(88.7)	
Yes	1320(8.7)	130(6.3)	839(8.4)	351(11.3)	

Chi-squared test was used

*BMI* body mass index

After an average follow-up of 12.5 years, 9655 deaths from all causes were identified. In the fully adjusted model (Table 3), all lifestyle factors, except for BMI, were significantly associated with an increased risk of all-cause mortality. Specifically, compared with the elderly individuals with a normal weight, those who were overweight had a significantly decreased hazards ratio (HR) for total death, while the elderly individuals who were underweight had a significantly elevated HR.

**Table 3:** Longitudinal associations of lifestyle-related factors with all-cause mortality

Lifestyle factors	Deaths No.	Participants, No.	Model 1	Model 2	Model 3
			HR(95%CI)	HR(95%CI)	HR(95%CI)
Smoking status					
Never	6367	9983	1	1	1
Former	1880	2709	1.13(1.07,1.20)	1.16(1.09,1.22)	1.15(1.09,1.22)
Current	1408	2471	1.08(1.02,1.15)	1.07(1.00,1.14)	1.07(1.00,1.14)
Alcohol consumption					
Never	6337	10,111	1	1	1
Former	1503	2422	0.98(0.92,1.03)	0.95(0.89,1.01)	0.95(0.89,1.01)
Current	1815	2630	1.11(1.05,1.17)	1.09(1.03,1.15)	1.09(1.03,1.15)
BMI, kg/m^2^					
< 18.5	95	131	3.03(2.47,3.72)	3.04(2.48,3.73)	3.01(2.45,3.69)
18.5- 23.9	4055	6580	1	1	1
24.0-27.9	4108	6370	0.94(0.90,0.99)	0.93(0.89,0.98)	0.93(0.89,0.98)
≥ 28.0	1397	2082	0.92(0.86,0.99)	0.91(0.85,0.98)	0.91(0.85,0.98)
Dietary diversity					
Poor	4736	6851	1	1	1
Good	4919	8312	0.92(0.88,0.96)	0.94(0.91,0.98)	0.94(0.90,0.98)
Regular exercise					
No	6951	10,318	1	1	1
Yes	2704	4845	0.80(0.76,0.84)	0.81(0.77,0.85)	0.81(0.77,0.85)

*BMI* body mass index

Model 1 was adjusted for age and sex

Model 2 was adjusted for age, sex, education level, marital status, place of residence and other lifestyle factors

Model 3 was further adjusted for physical comorbidities at baseline

The associations between lifestyle categories and total mortality risk are provided in Table 4. In comparison with participants with an unhealthy lifestyle, participants with intermediate and healthy lifestyle patterns had 10% (HR = 0.90, 95% CI = 0.85-0.96) and 22% (HR = 0.78, 95% CI = 0.72-0.83) lower risks of mortality from all causes, respectively, in the fully adjusted models. Not having a healthy lifestyle was related to a total PAR of 25.2% for all-cause mortality.

**Table 4:** Healthy lifestyle categories and risk of all-cause mortality

Lifestyle categories	Model 1	Model 2	Model 3
	HR (95% CI)	HR (95% CI)	HR (95% CI)
Unhealthy lifestyle (*n* = 2056 [13.6%])	1	1	1
Intermediate lifestyle (*n* = 9988[65.9%])	0.89(0.84,0.95)	0.90(0.85,0.96)	0.90(0.85,0.96)
Healthy lifestyle (*n* = 3119[20.5%])	0.76(0.71,0.82)	0.78(0.72,0.83)	0.78(0.72,0.83)
*P* value for trend	<.001	<.001	<.001
Total PAR^a^	26.6%	25.4%	25.2%

Model 1 was adjusted for age and sex

Model 2 was adjusted for age, sex, education level, marital status and place of residence

Model 3 was further adjusted for physical comorbidities at baseline

^a^Estimated by summing specific PARs of each exposure category from unhealthy to intermediate lifestyle using a healthy lifestyle group as the reference

In the subgroup analyses (eTables 1, 2, 3, 4, 5, 6, 7, 8, 9, 10 and 11), age, sex, education level, marital status and residence had significant interactions with lifestyle factors regarding the total mortality risk (all *P*_*-interaction*_ < .05). Significant differences were detected among individuals with various demographic characteristics (all *P*_*-heterogeneity*_ < .05), stronger associations were observed among younger, female, married, more educated individuals and those living in urban areas. Compared with the general population, no statistical significance was observed among individuals suffering from hypertension, heart disease, diabetes or cancer (*P*_*-interaction*_ > .05), while the association were stronger among participants with cerebrovascular disease (*P*_*-interaction*_ < .05). We stratified the participants into three subgroups according to the severity of physical comorbidities, and interactions were also observed between the severity of physical comorbidities and lifestyle factors concerning the total mortality risk (*P*_*-interaction*_ < .001). A healthier lifestyle was associated with a lower risk of all-cause mortality among individuals with different severities of physical comorbidities, whereas the associations were stronger among those with fatal diseases (*P*_*-heterogeneity*_ < .001) (eTable 6).

Several sensitivity analyses were carried out to investigate the potential bias existing in this study, with findings similar to the main findings (eTables 12, 13, 14 and 15). Interestingly, after alternatively subtracting one component at a time from the original healthy lifestyle score, the results remained largely compatible (eFigure1).

**Fig. 1:**
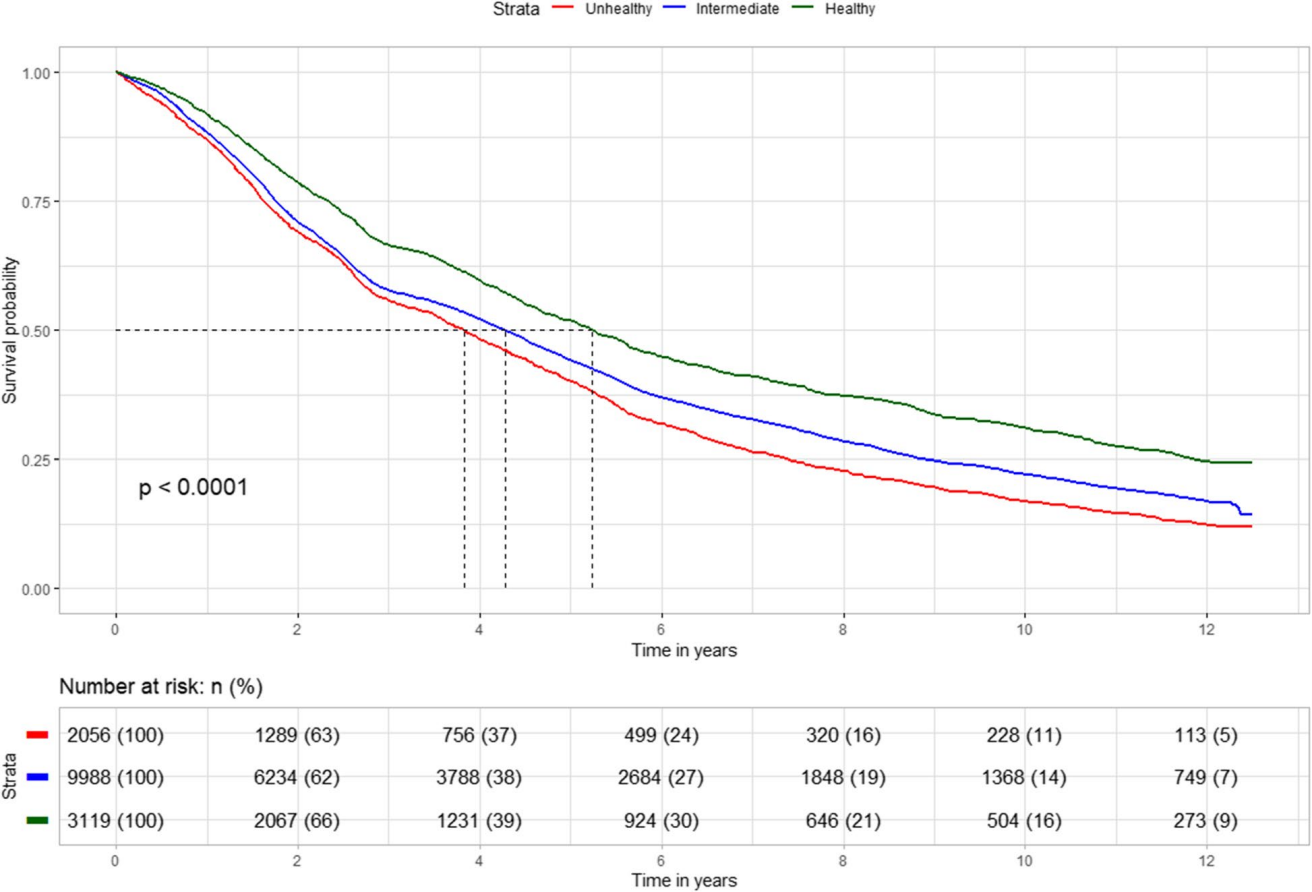
Survival of older individuals with unhealthy, intermediate and healthy lifestyles. The log-rank test was used

Figure 1 displays the cumulative survival estimates from the Cox proportional hazards regression model. In general, a clear pattern of higher survival in the individuals with a healthy lifestyle and lower survival in the individuals with an unhealthy lifestyle was observed (*P* < .001).

## Discussion

We found that healthy lifestyle factors, including a normal BMI, regular exercise, a more diverse diet, never smoking and never drinking, were significantly and independently associated with a lower risk of total mortality. Healthy lifestyle scores, representing joint measures of these five factors, were significantly associated with a lower risk of mortality as the number of healthy factors increased. The associations remained significant across all participants with different severities of physical comorbidities, and the associations were stronger among the female, younger, and married individuals, and those living in urban areas and with a higher education.

Consistent with previous studies, our findings suggested a significant inverse association between the number of healthy lifestyle factors and all-cause mortality. However, we found that participants with healthy lifestyle patterns had a 22% lower risk of all-cause mortality, which was slightly lower than the results of previous studies. Two meta-analyses reported that a combination of multiple healthy behaviors was associated with 55 and 66% reductions in all-cause mortality [11, 12]. This phenomenon might be explained by the widely variable characteristics of the study populations (e.g., age, sex composition, race or ethnicity), different definitions of healthy lifestyles, different study designs (e.g., follow-up durations), or different statistical analysis methods (e.g., adjusted covariates) [33, 34]. For instance, most previous studies included white adults in the general population, while our study focused on the elderly Chinese population. In addition, in the subgroup analysis stratified by age, the results also showed a strong association in younger elderly individuals, which might help explain this variability to some extent.

The lifestyle factors chosen in this study included smoking, drinking, BMI, dietary diversity and exercise as these factors are modifiable and easy to assess and interpret [35–38]. Our findings for the independent effects of tobacco smoking, alcohol consumption, regular exercise participation, and high food diversity on the risk of total mortality were in line with those of previous reports [16, 21, 39]. Nevertheless, the relationship between BMI and total death remains controversial [15, 40]. Several large cohort studies have shown both overweight and underweight to be independent predictors of an increased risk for mortality [40], while our findings indicated that only underweight individuals were subject to a higher risk of mortality, and overweight elderly individuals had a lower risk of death than elderly individuals with normal weight. The results of a previous meta-analysis including an elderly population support our results; that study also found that overweight individuals had a lower risk of all-cause mortality [41]. Possible explanations include the protective effects of cardiovascular metabolism by increased body fat, earlier presentation of overweight patients to medical facilities and earlier receipt of optimal medical treatment [42–45]. Moreover, since an underweight status might indicate a poor health condition and individuals who were underweight may die soon after the start of the follow-up period, we excluded participants who died within the first year and those with potentially fatal diseases at baseline, and the findings were still similar.

Our results show a stronger relationship between joint lifestyle factors and total mortality among the younger elder, women, and those married, literate and living in urban regions. A large UK cohort supports the findings of our study to some degree as the authors found that the association of joint lifestyle factors was stronger in younger than older individuals [46]. However, a meta-analysis contrasted our findings and found that the associations were largely consistent among populations from different socioeconomic backgrounds [12]. Individuals’ socioeconomic status could affect their access to multiple resources (e.g. wealth, knowledge, and healthcare services), and those with a higher socioeconomic status were more likely to engage in healthier lifestyles. A systematic review indicated that 20 to 30% of the lifestyle factors in health outcomes were attributed to socioeconomic inequity [47]. The exact explanations for these inconsistent findings remain unclear, and more investigations are needed to examine the complicated relationship among lifestyles, socioeconomic status, total deaths and other outcomes.

Our study highlights the important question of whether individuals with diseases can benefit from the adoption of healthy behaviors. Among participants with hypertension, diabetes, heart disease or cancer at baseline, relationships between lifestyle factors and risk of all-cause mortality were similar to those in the general population. Although the associations were stronger among these patients, no significant differences were detected in the heterogeneity tests. We stratified the participants by the severity of five chronic diseases and found a stronger association between lifestyle factors and total mortality among those with fatal diseases. This finding suggests that the severity of disease and corresponding treatment are also important prognosis predictors among patients, strengthening the effects of lifestyle factors. The substantial reductions in the total mortality risk further support the results of previous studies and recommendations, indicating that lifestyle modifications are meaningful for population with disease [48–52].

The main strengths of this study were the large sample size, prospective design and large number of accumulated deaths, which improved the generalizability of our findings. Additionally, several sensitivity analyses were conducted to further complement the main analyses and show the robustness of our results. Finally, we calculated the PARs of lifestyle factors on total mortality, and the results indicated that 25.2% of deaths were attributed to not having a healthy lifestyle. These results are easy to interpret and can provide public health guidance.

Nonetheless, this study also has several limitations. First, the lifestyle factors were mainly self-reported, and even though self-reporting is common in most large epidemiological studies, measurement errors are inevitable. In addition, data concerning deaths due to specific causes were unavailable, limiting the analyses of the associations between lifestyle factors and mortality due to specific causes among elderly individuals. Thus, future studies investigating the impacts of lifestyle factors on total deaths and other health outcomes are warranted. Moreover, approximately 25% of the participants were lost to follow-up. Nevertheless, most of the individuals were lost to follow-up due to city construction or moving to another living place, and this may not lead to substantial bias. And after we excluded those lost to follow-up, the repeating analysis results were similar to the overall results (not presented). Second, healthy lifestyle scores were calculated assuming that each of the factors was equally weighted. However, the factors included in this study may interrelate and interact with each other; for instance, participants who performed regular exercise were more likely to have normal weight, which may affect the precision of the estimates. However, after we alternately removed one component at a time from the initial healthy lifestyle scores, the tests showed compatibility across all cases and the overall results. Third, due to the presence of subclinical or clinical diseases, there might be some potential reverse causation bias. To address this concern, we excluded participants with potentially fatal diseases at baseline, those with underweight, those aged ≥85 years (who had a higher risk of death during the follow-up period), and those who died within the first year of follow-up. Repeated analyses in those subgroups were still robust. Last, the CLHLS included extensive data on demographic characteristics, lifestyle factors and chronic physical comorbidities, enabling the performance of analyses for multiple lifestyle variables with multivariate adjustments, which most previous studies did not control for, yet the relationship between mental health and mortality risk was not assessed and residual cofounding may still be present. Future investigations were needed to explore the effect of mental disorders on mortality.

## Conclusions

In conclusion, this study suggests that a substantial reduction in total death could be achieved by the adoption of a healthier lifestyle based on five factors. The impacts of combined lifestyle factors on the reduction in total mortality were observed among those with and without preexisting chronic diseases. Given the aging population and increasing limitation of medical resources in China, cost-effective lifestyle interventions (such as raising public awareness and launching public education programs), which are affordable, may be developed to respond to the challenges posed by population aging.

## Supplementary Information

**Additional file 1: eFigure 1.** Hazards ratio associated with 1-unit increase in the healthy lifestyle score and after subtracting of one factor at a time. **eTable 1.** Subgroup analysis between lifestyle categories and risk of total mortality stratified by age. **eTable 2.** Subgroup analyses between lifestyle categories and risk of total mortality stratified by sex. **eTable 3.** Subgroup analyses between lifestyle categories and risk of total mortality stratified by place of residence. **eTable 4.** Subgroup analyses between lifestyle categories and risk of total mortality stratified by education level. **eTable 5.** Subgroup analyses between lifestyle categories and risk of total mortality stratified by marital status. **eTable 6.** Subgroup analyses between lifestyle categories and risk of total mortality stratified by chronic disease status at baseline. **eTable 7.** Subgroup analyses between lifestyle categories and risk of total mortality stratified by hypertension status at baseline. **eTable 8.** Subgroup analyses between lifestyle categories and risk of total mortality stratified by diabetes status at baseline. **eTable 9.** Subgroup analyses between lifestyle categories and risk of total mortality stratified by heart disease status at baseline. **eTable 10.** Subgroup analyses between lifestyle categories and risk of total mortality stratified by cerebrovascular disease status at baseline. **eTable 11.** Subgroup analyses between lifestyle categories and risk of total mortality stratified by cancer status at baseline. **eTable 12.** Sensitivity analyses by excluding participants aged ≥85 years, *n* = 6687. **eTable 13.** Sensitivity analyses by excluding participants died within the first year, *n* = 13,687. **eTable 14.** Sensitivity analyses by excluding participants with potentially fatal chronic diseases at baseline, *n* = 11,945. **eTable 15.** Sensitivity analyses by excluding underweight (BMI < 18.5 kg/m^2^) participants, *n* = 15,032.

## Data Availability

The data that support the findings of this study are available from the Chinese Longitudinal Healthy Longevity Survey (CLHLS) group, but restrictions apply to the availability of these data, which were used under license for current study, and so are not publicly available. Data are however available from the authors upon reasonable request and with the permission of the CLHLS group (Chinese Longitudinal Healthy Longevity Survey (CLHLS) – Duke Aging Center).

## Abbreviations

CLHLS: Chinese Longitudinal Healthy Longevity Survey
BMI: Body mass index
NCDs: Non-communicable diseases
SD: Standard deviation
PAR: Population attributable risk
SPSS: Statistical Package for the Social Sciences
HRs: Hazards ratios
CI: Confidence interval
