# Supplementary material for: Combined impact of lifestyle-related factors on total mortality among the elder Chinese: a prospective cohort study
Source: BMC Geriatr. 2022 Apr 14;22:325. doi: 10.1186/s12877-022-02982-z (PMC9009055; doi:10.1186/s12877-022-02982-z)
Supplement: Supplementary file 1 — Additional file 1: eFigure 1. Hazards ratio associated with 1-unit increase in the healthy lifestyle score and after subtracting of one factor at a time. eTable 1. Subgroup analysis between lifestyle categories and risk of total mortality stratified by age. eTable 2. Subgroup analyses between lifestyle categories and risk of total mortality stratified by sex. eTable 3. Subgroup analyses between lifestyle categories and risk of total mortality stratified by place of residence. eTable 4. Subgroup analyses between lifestyle categories and risk of total mortality stratified by education level. eTable 5. Subgroup analyses between lifestyle categories and risk of total mortality stratified by marital status. eTable 6. Subgroup analyses between lifestyle categories and risk of total mortality stratified by chronic disease status at baseline. eTable 7. Subgroup analyses between lifestyle categories and risk of total mortality stratified by hypertension status at baseline. eTable 8. Subgroup analyses between lifestyle categories and risk of total mortality stratified by diabetes status at baseline. eTable 9. Subgroup analyses between lifestyle categories and risk of total mortality stratified by heart disease status at baseline. eTable 10. Subgroup analyses between lifestyle categories and risk of total mortality stratified by cerebrovascular disease status at baseline. eTable 11. Subgroup analyses between lifestyle categories and risk of total mortality stratified by cancer status at baseline. eTable 12. Sensitivity analyses by excluding participants aged ≥85 years, n = 6687. eTable 13. Sensitivity analyses by excluding participants died within the first year, n = 13,687. eTable 14. Sensitivity analyses by excluding participants with potentially fatal chronic diseases at baseline, n = 11,945. eTable 15. Sensitivity analyses by excluding underweight (BMI < 18.5 kg/m2) participants, n = 15,032. [file 12877_2022_2982_MOESM1_ESM.docx]

**Combined impact of lifestyle-related factors on total mortality among the elder Chinese: A Prospective cohort study**

**Supplementary material**

| **No.** | **Contents** | **Page No.** |
| --- | --- | --- |
| 1 | Supplementary figure | 1 |
| 2 | Supplementary tables | 2-16 |

**Supplementary figure**


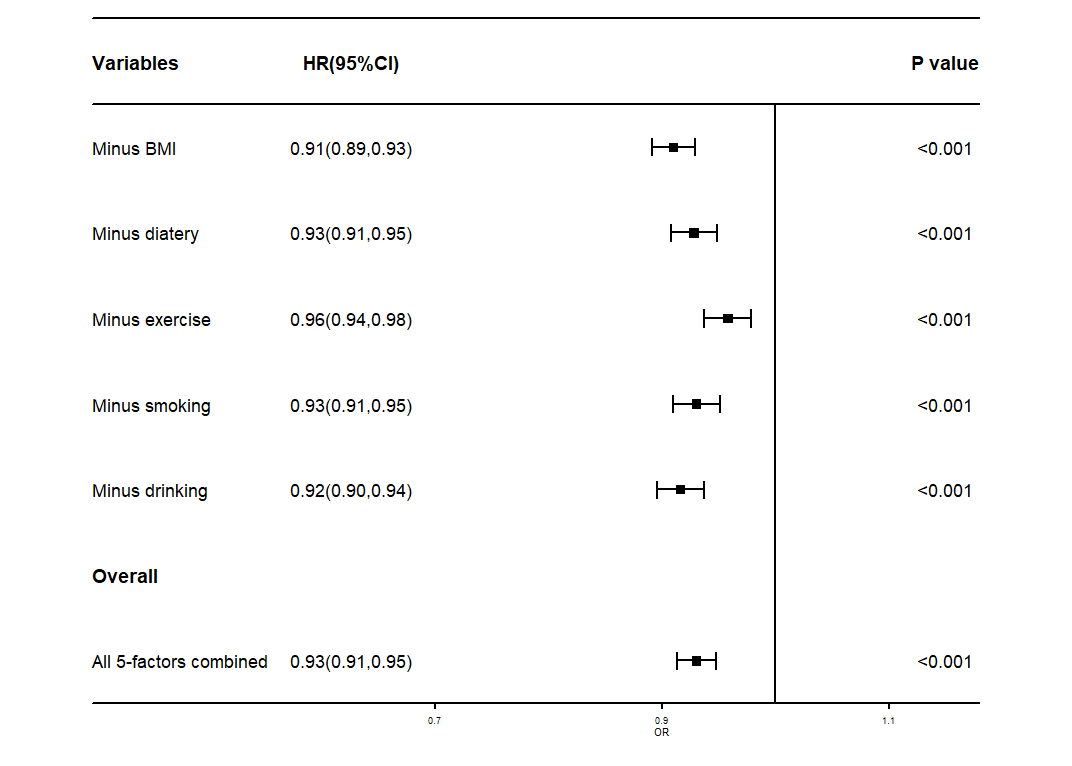


**eFigure 1. Hazards ratio associated with 1-unit increase in the healthy lifestyle score and after subtracting of one factor at a time**

**Supplementary tables**

**eTable1. Subgroup analysis between lifestyle categories and risk of total mortality stratified by age.**

|  | **HR(95%CI)** | **P-value** | **p_-trend_** | **p_-heterogeneity_** | **p_-interaction_** |
| --- | --- | --- | --- | --- | --- |
| **65~79** |  |  | <.001 | <.001 | .010 |
| Unhealthy lifestyle | 1 |  |  |  |  |
| Intermediate lifestyle | 0.82(0.72,0.94) | .004 |  |  | .058 |
| Healthy lifestyle | 0.68(0.58,0.80) | <.001 |  |  | .003 |
| **80~94** |  |  | <.001 |  |  |
| Unhealthy lifestyle | 1 |  |  |  |  |
| Intermediate lifestyle | 0.90(0.83,0.98) | .015 |  |  |  |
| Healthy lifestyle | 0.76(0.69,0.85) | <.001 |  |  |  |
| **~95** | 1 |  | .006 |  |  |
| Unhealthy lifestyle |  |  |  |  |  |
| Intermediate lifestyle | 0.95(0.87,1.05) | .321 |  |  |  |
| Healthy lifestyle | 0.84(0.75,0.95) | .006 |  |  |  |

Model was adjusted for age, sex, education level, marital status, place of residence and physical comorbidities.

**eTable 2. Subgroup analyses between lifestyle categories and risk of total mortality stratified by sex**

|  | **HR(95%CI)** | **P-value** | **p_-trend_** | **p_-heterogeneity_** | **p_-interaction_** |
| --- | --- | --- | --- | --- | --- |
| **Female** |  |  | <.001 | .011 | .008 |
| Unhealthy lifestyle | 1 |  |  |  |  |
| Intermediate lifestyle | 0.91(0.84,0.99) | .020 |  |  | .961 |
| Healthy lifestyle | 0.72(0.65,0.79) | <.001 |  |  | .026 |
| **Male** |  |  | .001 |  |  |
| Unhealthy lifestyle | 1 |  |  |  |  |
| Intermediate lifestyle | 0.91(0.84,0.98) | .016 |  |  |  |
| Healthy lifestyle | 0.84(0.76,0.93) | .001 |  |  |  |

Model was adjusted for age, education level, marital status, place of residence and physical comorbidities.

**eTable 3. Subgroup analyses between lifestyle categories and risk of total mortality stratified by place of residence**

|  | **HR(95%CI)** | **P-value** | **p_-trend_** | **p_-heterogeneity_** | **p_-interaction_** |
| --- | --- | --- | --- | --- | --- |
| **Urban** |  |  | <.001 | .039 | .043 |
| Unhealthy lifestyle | 1 |  |  |  |  |
| Intermediate lifestyle | 0.89(0.81,0.99) | .023 |  |  | .810 |
| Healthy lifestyle | 0.72(0.65,0.81) | <.001 |  |  | .043 |
| **Rural** |  |  | <.001 |  |  |
| Unhealthy lifestyle | 1 |  |  |  |  |
| Intermediate lifestyle | 0.91(0.84,0.97) | .006 |  |  |  |
| Healthy lifestyle | 0.84(0.76,0.92) | <.001 |  |  |  |

Model was adjusted for age, sex, education level, marital status, and physical comorbidities.

**eTable 4. Subgroup analyses between lifestyle categories and risk of total mortality stratified by education level**

|  | **HR(95%CI)** | **P_-trend_** | **P_-heterogeneity_** | **P_-interaction_** |
| --- | --- | --- | --- | --- |
| **Literacy** |  | <.001 | <.001 | <.001 |
| Unhealthy lifestyle | 1 |  |  |  |
| Intermediate lifestyle | 0.87(0.78,0.96) |  |  | .302 |
| Healthy lifestyle | 0.66(0.59,0.74) |  |  | <.001 |
| **Illiteracy** |  | .002 |  |  |
| Unhealthy lifestyle | 1 |  |  |  |
| Intermediate lifestyle | 0.92(0.86,0.99) |  |  |  |
| Healthy lifestyle | 0.86(0.79,0.95) |  |  |  |

Model was adjusted for age, sex, marital status, place of residence and physical comorbidities.

**eTable 5. Subgroup analyses between lifestyle categories and risk of total mortality stratified by marital status**

|  | **HR(95%CI)** | **P-value** | **p_- trend_** | **P_-heterogeneity_** | **p_-interaction_** |
| --- | --- | --- | --- | --- | --- |
| **Married** |  |  | <.001 | .045 | .044 |
| Unhealthy lifestyle | 1 |  |  |  |  |
| Intermediate lifestyle | 0.80(0.70,0.90) | <.001 |  |  | .023 |
| Healthy lifestyle | 0.67(0.58,0.77) | <.001 |  |  | .020 |
| **Widowed and others** |  |  | <.001 |  |  |
| Unhealthy lifestyle | 1 |  |  |  |  |
| Intermediate lifestyle | 0.93(0.87,0.99) | .032 |  |  |  |
| Healthy lifestyle | 0.81(0.74,0.88) | <.001 |  |  |  |

Model was adjusted for age, sex, education level, place of residence and physical comorbidities.

**eTable 6. Subgroup analyses between lifestyle categories and risk of total mortality stratified by chronic disease status at baseline.**

|  | **HR(95%CI)** | **p-value** | **p_-trend_** | **p_- heterogeneity_** | **p_-interaction_** |
| --- | --- | --- | --- | --- | --- |
| **The elder with fatal diseases** |  |  | <.001 | <.001 | <.001 |
| Unhealthy lifestyle | 1 |  |  |  |  |
| Intermediate lifestyle | 0.89(0.78,1.03) | .111 |  |  |  |
| Healthy lifestyle | 0.74(0.62,0.87) | <.001 |  |  |  |
| **The elder with hypertension and diabetes at baseline** |  |  |  |  |  |
| Unhealthy lifestyle | 1 |  |  |  | .003 |
| Intermediate lifestyle | 0.94(0.78,1.14) | .512 |  |  | <.001 |
| Healthy lifestyle | 0.77(0.61,0.96) | .022 |  |  |  |
| **Healthy individuals** |  |  | <.001 |  |  |
| Unhealthy lifestyle | 1 |  |  |  |  |
| Intermediate lifestyle | 0.90(0.84,0.96) | .002 |  |  | .219 |
| Healthy lifestyle | 0.79(0.72,0.86) | <.001 |  |  | .264 |

Model was adjusted for age, sex, education level, marital status, and place of residence.

**eTable 7 Subgroup analyses between lifestyle categories and risk of total mortality stratified by hypertension status at baseline.**

| **Hypertension** | **HR(95%CI)** | **P-value** | **P_-trend_** | **P_-heterogeneity_** | **p_-interaction_** |
| --- | --- | --- | --- | --- | --- |
| **yes** |  |  | <.001 | .534 | .540 |
| Unhealthy lifestyle | 1 |  |  |  |  |
| Intermediate lifestyle | 0.87(0.76,0.99) | .028 |  |  | .487 |
| Healthy lifestyle | 0.73(0.62,0.85) | <.001 |  |  | .266 |
| **no** |  |  | <.001 |  |  |
| Unhealthy lifestyle | 1 |  |  |  |  |
| Intermediate lifestyle | 0.91(0.86,0.97) | .004 |  |  |  |
| Healthy lifestyle | 0.79(0.73,0.86) | <.001 |  |  |  |

Model was adjusted for age, sex, education level, marital status, place of residence and physical comorbidities (diabetes, heart disease, Cerebrovascular disease and cancer).

**eTable 8 Subgroup analyses between lifestyle categories and risk of total mortality stratified by diabetes status at baseline.**

| **Diabetes** | **HR(95%CI)** | **P-value** | **P_-trend_** | **P_-heterogeneity_** | **p_-interaction_** |
| --- | --- | --- | --- | --- | --- |
| **yes** |  |  | .032 | .973 | .937 |
| Unhealthy lifestyle | 1 |  |  |  |  |
| Intermediate lifestyle | 0.91(0.75,1.10) | .304 |  |  | .583 |
| Healthy lifestyle | 0.80(0.64,0.99) | .044 |  |  | .601 |
| **no** |  |  | <.001 |  |  |
| Unhealthy lifestyle | 1 |  |  |  |  |
| Intermediate lifestyle | 0.90(0.85,0.96) | .001 |  |  |  |
| Healthy lifestyle | 0.77(0.71,0.83) | <.001 |  |  |  |

Model was adjusted for age, sex, education level, marital status, place of residence and physical comorbidities (hypertension, heart disease, Cerebrovascular disease and cancer).

**eTable 9 Subgroup analyses between lifestyle categories and risk of total mortality stratified by heart disease status at baseline.**

| **Heart diseases** | **HR(95%CI)** | **P-value** | **p_-trend_** | **P_-heterogeneity_** | **p_-interaction_** |
| --- | --- | --- | --- | --- | --- |
| **yes** |  |  | <.001 | .304 | .297 |
| Unhealthy lifestyle | 1 |  |  |  |  |
| Intermediate lifestyle | 0.83(0.71,0.97) | .021 |  |  | .184 |
| Healthy lifestyle | 0.71(0.59,0.85) | <.001 |  |  | .127 |
| **no** |  |  | <.001 |  |  |
| Unhealthy lifestyle | 1 |  |  |  |  |
| Intermediate lifestyle | 0.92(0.86,0.97) | .004 |  |  |  |
| Healthy lifestyle | 0.79(0.73,0.85) | <.001 |  |  |  |

Model was adjusted for age, sex, education level, marital status, place of residence and physical comorbidities (diabetes, hypertension, Cerebrovascular disease and cancer).

**eTable 10. Subgroup analyses between lifestyle categories and risk of total mortality stratified by cerebrovascular disease status at baseline.**

| **Cerebrovascular disease** | **HR(95%CI)** | **P-value** | **P_- trend_** | **P_-heterogeneity_** | **P_-interaction_** |
| --- | --- | --- | --- | --- | --- |
| **yes** |  |  | .032 | .910 | <.001 |
| Unhealthy lifestyle | 1 |  |  |  |  |
| Intermediate lifestyle | 0.97(0.82,1.16) | .759 |  |  | <.001 |
| Healthy lifestyle | 0.83(0.67,1.01) | .066 |  |  | .009 |
| **no** |  |  | <.001 |  |  |
| Unhealthy lifestyle | 1 |  |  |  |  |
| Intermediate lifestyle | 0.90(0.84,0.95) | <.001 |  |  |  |
| Healthy lifestyle | 0.77(0.71,0.83) | <.001 |  |  |  |

Model was adjusted for age, sex, education level, marital status, place of residence and physical comorbidities (diabetes, hypertension, heart disease and cancer).

**eTable 11. Subgroup analyses between lifestyle categories and risk of total mortality stratified by cancer status at baseline.**

| **Cancer** | **HR(95%CI)** | **P-value** | **P_-trend_** | **P_-heterogeneity_** | **P_-interaction_** |
| --- | --- | --- | --- | --- | --- |
| **yes** |  |  | .003 | .526 | .494 |
| Unhealthy lifestyle | 1 |  |  |  |  |
| Intermediate lifestyle | 0.85(0.67,1.08) | .193 |  |  | .510 |
| Healthy lifestyle | 0.69(0.52,0.90) | .007 |  |  | .270 |
| **no** |  |  | <.001 |  |  |
| Unhealthy lifestyle | 1 |  |  |  |  |
| Intermediate lifestyle | 0.91(0.85,0.96) | .001 |  |  |  |
| Healthy lifestyle | 0.78(0.73,0.84) | <.001 |  |  |  |

Model was adjusted for age, sex, education level, marital status, place of residence and physical comorbidities (diabetes, hypertension, Cerebrovascular disease and heart disease).

**eTable 12. Sensitivity analyses by excluding participants aged ≥ 85 years, n= 6687.**

|  | **Model 1** | **Model 2** | **Model 3** |
| --- | --- | --- | --- |
|  | **HR (95% CI)** | **HR (95% CI)** | **HR (95% CI)** |
| Unhealthy lifestyle  (n = 849 [12.7%]) | 1 | 1 | 1 |
| Intermediate lifestyle  (n = 4225[63.2%]) | 0.82(0.74,0.91) | 0.83(0.75,0.92) | 0.83(0.75,0.92) |
| Healthy lifestyle  (n = 1613[24.1%]) | 0.68(0.61,0.77) | 0.71(0.63,0.81) | 0.71(0.63,0.81) |
| P value for trend | <.001 | <.001 | <.001 |

Model 1 was adjusted for age and sex.

Model 2 was adjusted for age, sex, education level, marital status.

Model 3 was further adjusted for physical comorbidities at baseline.

**eTable 13. Sensitivity analyses by excluding participants died within the first year, n= 13687**

|  | **Model 1** | **Model 2** | **Model 3** |
| --- | --- | --- | --- |
|  | **HR (95% CI)** | **HR (95% CI)** | **HR (95% CI)** |
| Unhealthy lifestyle  (n = 1812 [13.2%]) | 1 | 1 | 1 |
| Intermediate lifestyle  (n = 8970[69.0%]) | 0.89(0.84,0.95) | 0.90(0.85,0.96) | 0.90(0.85,0.96) |
| Healthy lifestyle  (n = 2905[21.2%]) | 0.77(0.71,0.83) | 0.78(0.73,0.85) | 0.79(0.73,0.85) |
| P value for trend | <.001 | <.001 | <.001 |

Model 1 was adjusted for age and sex.

Model 2 was adjusted for age, sex, education level, marital status.

Model 3 was further adjusted for physical comorbidities at baseline.

**eTable 14. Sensitivity analyses by excluding participants with potentially fatal chronic diseases at baseline, n=11945**

|  | **Model 1** | **Model 2** | **Model 3** |
| --- | --- | --- | --- |
|  | **HR (95% CI)** | **HR (95% CI)** | **HR (95% CI)** |
| Unhealthy lifestyle  (n = 1812 [13.2%]) | 1 | 1 | 1 |
| Intermediate lifestyle  (n = 8970[69.0%]) | 0.89(0.84,0.96) | 0.91(0.85,0.96) | 0.91(0.85,0.97) |
| Healthy lifestyle  (n = 2905[21.2%]) | 0.78(0.72,0.84) | 0.78(0.72,0.85) | 0.78(0.73,0.85) |
| P value for trend | <.001 | <.001 | <.001 |

Model 1 was adjusted for age and sex.

Model 2 was adjusted for age, sex, education level, marital status.

Model 3 was further adjusted for hypertension and diabetes at baseline.

**eTable 15. Sensitivity analyses by excluding underweight (BMI <18.5 kg/m^2^) participants, n= 15032**

|  | **Model 1** | **Model 2** | **Model 3** |
| --- | --- | --- | --- |
|  | **HR (95% CI)** | **HR (95% CI)** | **HR (95% CI)** |
| Unhealthy lifestyle  (n = 1812 [13.2%]) | 1 | 1 | 1 |
| Intermediate lifestyle  (n = 8970[69.0%]) | 0.90(0.85,0.95) | 0.91(0.86,0.96) | 0.91(0.86,0.96) |
| Healthy lifestyle  (n = 2905[21.2%]) | 0.77(0.72,0.83) | 0.79(0.73,0.84) | 0.79(0.73,0.85) |
| P value for trend | <.001 | <.001 | <.001 |

Model 1 was adjusted for age and sex.

Model 2 was adjusted for age, sex, education level, marital status.

Model 3 was further adjusted for physical comorbidities at baseline.
